# Up-cycling the dental waste materials: Going green in dentistry

**DOI:** 10.6026/9732063002001124

**Published:** 2024-09-30

**Authors:** Kranti Rajesh Khadse, Swati Priya, Rana K. Varghese, Malwika Sisodia, Naveen Kumar Gupta, Anita Chandrakar

**Affiliations:** 1 Department of Conservative Dentistry and Endodontics, New Horizon Dental College and Research Institute, Sakri, Bilaspur, Chhattsigarh, India

**Keywords:** Up-cycling, reduce, reuse, dental waste material

## Abstract

The depletion of natural resources due to the extensive use of various materials in dentistry is a growing concern. A significant contributor to this issue is the lack of recycling practices, leading to the continuous exploitation of these resources. This research aims to explore simple yet effective scientific techniques to up-cycle common dental materials while maintaining their properties. The focus of modern practices should align with the 3R's-Reduce, Reuse, and Recycle. In dental institutions and clinics, materials like dental plaster and dental stone, extensively used for pouring impressions, are completely discarded as waste. This study involves collecting these waste products and evaluating their properties for potential reuse. Additionally, endodontic materials such as stainless steel hand files and rotary Ni-Ti files, typically discarded, can be recycled in metallurgical departments and repurposed into custom-made cast posts and titanium posts, respectively. The wisdom tooth crush technique is explored for its application as autologous graft material in filling bone defects. Discarded alginate impressions, widely used in Prosthodontics, are heated and repurposed as a denture polishing agent and as a fertilizer in agriculture. Excess glass ionomer cement, a common restorative material, can be repurposed into polishing cones and powders, or used as a polishing agent with rubber cups and buffs. Modeling wax, predominantly used in occlusal rim fabrication, can be collected and purified, with 90% of it recoverable without compromising its properties. Finally, discarded dental burs can be repurposed to splint implant impression copings, aiding in the accurate transfer of orientation, a critical step in implant prosthodontics. This study advocates for the adoption of recycling practices in dental colleges and among practitioners to manage and reuse the waste generated in the field. By doing so, the treatment cost can be reduced by 30-40%, and natural resources can be conserved, contributing to the preservation of the Earth's resources.

## Background:

The depletion of natural resources is a growing concern due to the extensive use of various materials in different fields of dentistry. A significant factor contributing to this issue is the lack of recycling, which exacerbates the exploitation and depletion of these resources. Dental treatments require a wide range of materials and equipment, often fabricated from metallic and non-metallic resources derived from nature, presenting potential environmental challenges. Dentistry, like many other fields in the medical industry, generates substantial amounts of environmentally "unfriendly waste" that significantly impact the environment. To mitigate this, the four R's-Reduce, Reuse, Recycle, and Rethink-are essential for reducing environmental harm and promoting sustainability [1, 2, 3- 4]. In dentistry, efforts have been made to recycle and reuse certain materials. This work aims to identify simple scientific techniques for recycling and reusing dental waste materials without compromising their properties [1, 2, 3- 4].

## Materials and Methods:

This study explores the recycling and reuse of various dental materials commonly discarded in dental practices. **Dental plaster (Type II) and dental stone (Type III) ** are widely used for pouring impressions to create casts. These materials, derived from gypsum, are processed through calcination to produce either plaster or dental stone, depending on the conditions. Discarded dental casts are first disinfected, then subjected to dry or wet calcination, and finally ground into powder for reuse in making new casts. **Rotary NiTi and stainless steel files**, essential in endodontic, are typically discarded after use. These files are sterilized and subjected to chemical processes to separate their components. For example, NiTi files are treated in concentrated hydrochloric acid to isolate titanium, which is then melted and repurposed into custom-made posts. Stainless steel files undergo a similar process and are melted down to create pellets for reuse. In another innovative approach, **extracted wisdom teeth** are repurposed as autologous bone graft material. The teeth, preserved in saline, are crushed and used to fill bone defects during surgery, taking advantage of their osteoconductive, osteoinductive, and osteogenic properties. **Alginate impression material**, commonly used in dental impressions, is recycled by heating the used material to extract diatomite, a key component. The diatomite is then repurposed as a polishing agent or as fertilizer. **Glass ionomer cement (GIC)**, often mixed in excess during restorative procedures, is disinfected, ground into powder and reused as a polishing agent for tooth-coloured fillings. Similarly, **dental waxes**, used extensively in prosthetic fabrication, are recycled by melting and re-forming them into new wax sheets, maintaining their properties without any significant degradation. Finally "dental burs" which are typically discarded after clinical use, are sterilized and repurposed in implant prosthodontics to splint impression copings. This method overcomes the limitations of traditional splinting techniques, reducing material shrinkage and improving the accuracy of implant impressions. By implementing these recycling practices, dental institutions can significantly reduce waste, lower treatment costs, and contribute to environmental sustainability.

## Results:

By recycling the above all materials together in every dental college of India (approx. 329 dental colleges), there will be huge revenue generated (Table 1 & Table 2). Although studies on the recycling and reuse of dental waste materials are limited, a questionnaire study was conducted in all dental colleges of Chhattisgarh and private clinics in Bilaspur, Chhattisgarh. The study aimed to assess the attitude and knowledge of dental students and practitioners regarding waste management and the potential for up-cycling dental materials. The survey was distributed to 3,500 students and private clinics, with responses received from 800 students in dental colleges and 100 private practitioners [1].

## Discussion:

The questionnaire included both attitude-based and knowledge-based questions to gauge awareness and interest in recycling practices. The study revealed that while many students and practitioners are aware of dental waste management, there is a significant gap in knowledge regarding the up-cycling and reuse of dental materials. For example, 96% of respondents were aware of waste management practices, but few were knowledgeable about the specific techniques for recycling materials like gypsum plaster, NiTi files, or dental wax [2]. This indicates a need for increased education and training in this area, which could be addressed through voluntary programs designed to enhance and update knowledge about sustainable practices in dentistry. The research also explored the practical aspects of recycling various dental materials. For instance, the study found that recycled gypsum plaster retains chemical characteristics similar to commercial plaster, despite differences in fineness, particle size distribution and bulk unit weight [3]. This suggests that gypsum waste can be recycled multiple times without losing its original properties, though more studies are needed to refine the process and ensure consistency in the quality of the recycled material. The study also highlighted the issue of instrument separation, particularly with stainless steel hand files and NiTi rotary files, which contribute to material wastage. While previous studies have shown that NiTi instruments are not more fragile than stainless steel counterparts, the recycling of these broken files remains unexplored and warrants further investigation [4, 5, 6-7]. Additionally, the study examined the potential for reusing extracted wisdom teeth as autologous graft material. This technique requires minimal investment, as it uses commonly available tools like a bone crusher and mallet. The research emphasizes the advantages of this approach, such as the preservation of dental pulp cells, which play a crucial role in bone formation. Moreover, the study explored the recycling of alginate impression material, particularly its diatomite content, as a polishing abrasive. Although effective, the handling of diatomite must be done with care due to potential health concerns [8]. GIC, widely used in restorative procedures, can be repurposed into polishing cones and powders, while dental wax, often discarded after fabrication processes, can be recollected, purified, and reshaped into new sheets. These practices not only reduce material waste but also generate additional revenue for dental institutions. However, further research is needed to explore other potential applications, such as using recycled GIC in air abrasive systems or reusing dental wax in more complex dental appliances [8]. The study also highlighted the economic benefits of recycling and reusing dental materials. For example, the reuse of excess glass ionomer cement (GIC) as a polishing agent or for other dental applications can lead to significant cost savings. In a dental college setting, where large quantities of GIC are used and often discarded, the ability to reclaim and repurpose this material could reduce expenditures by a substantial margin. Similarly, the reuse of dental wax, commonly used in the fabrication of occlusal rims and other dental appliances, can help institutions save on material costs while also reducing environmental impact. By implementing these recycling practices, dental colleges could not only lower their operational costs but also promote sustainability within the industry [9, 10-11]. The environmental implications of these recycling practices cannot be overstated. Dentistry, like many other healthcare fields, contributes to a significant amount of waste that is not only environmentally unfriendly but also difficult to manage. By adopting practices that focus on the reuse and recycling of dental materials, the industry can reduce its environmental footprint and contribute to global sustainability efforts. The study's findings suggest that with proper education and infrastructure, dental practitioners and students can play a pivotal role in reducing waste and conserving natural resources. The move towards a more sustainable practice not only benefits the environment but also enhances the public image of the dental profession as one that is conscious of its ecological responsibilities [12]. However, the study also identified several challenges that need to be addressed to make these practices more widespread. One of the main issues is the lack of awareness and training among dental students and practitioners regarding the specific methods for recycling and reusing dental materials. While many are aware of general waste management practices, the intricacies of up-cycling materials such as NiTi files, alginate impression materials, and dental waxes are less well-known. To overcome these challenges, the study recommends incorporating waste management and recycling techniques into the dental curriculum, ensuring that future dental professionals are equipped with the knowledge and skills necessary to implement these practices in their clinical work. Additionally, further research is needed to refine the recycling processes and explore new applications for recycled dental materials, ensuring that these practices are both economically viable and effective in preserving material quality [13, 14].

## Conclusion:

Up-cycling some of these dental waste materials, there will be an additional income generated to all the dental colleges which will in-turn enable the poor people to take cost effective treatment. The total treatment cost could be reduced up-to 30-40%. Large quantity of natural resources can be saved from getting depleted by conserving these resources. So, our main motto through this study is to make the dental students undergoing training in colleges and dental practitioners aware of this magnanimous issue of appropriate waste management and reuse of waste generated by them.

## Figures and Tables

**Table 1:** Attitude based Questions

**Attitude Based Questions**			
**Question**	**Yes (%)**	**No (%)**	**Don't Know (%)**
Are you aware of waste management of Dental Materials?	96.1	3.9	-
Will you be interested to attend voluntary programs that enhance and upgrade your knowledge about waste management?	96.1	3.9	
Do you think improper waste management can be hazardous to health?	100	0	
Do you have any idea about re-use of waste dental materials	54.9	45.1	
Do you think it is important to segregate Dental waste from general waste?	100	0	
Do you think re-use of waste Dental Materials is economical and beneficial?	82.4	17.6	
Are you aware about use of extracted human teeth?	86.3	13.7	
Do you think re-use of dental waste and handling should be a part of curriculum?	98	2	

**Table 2:** Knowledge based Questions

**Knowledge based questions**		
**Question**		
Whose responsibility is the management of dental waste?	Dentist/Auxiliary	98
	Patient	2
	No one	0
Alginate impression can be re-used as?	Can't re use	25.5
	Fertilizer	23.5
	Don't know	51
Can we use extracted teeth as an auto graft material?	Yes	66.7
	No	7.8
	Don't Know	25.5
Which types of file frequently break with you?	Rotary	49
	Hand	51
Which alloy of manufacture is frequently broken?	Stainless steel	64.7
	Ni Ti	35.3
Do you think excess mixed GIC can be re-used?	Yes	25.5
	No	56.9
	Don't Know	17.6
Dental stone cast can be re-used as?	Can't re use it	21.6
	As dental powder	54.9
	Don't know	23.5
Re-use of broken Endodontic file as?	Disposed	74.5
	Re used	13.7
	Don't know	11.8
Do you think recycling of dental waste will generate extra income to dental college	Yes	47.1
	No	11.8
	May be	41.1
Can we re-use dental waxes used in occlusal rim fabrication and	Yes	68.6
	No	15.7
	Don't Know	15.7
Can we re-use waste dental amalgam?	Yes	23.5
	No	54.9
	Don't Know	21.6
How many GIC and Amalgam restorations do you do in a month?	More than 50	33.3
	More than 100	9.8
	Not estimated	56.9
How many patients are done using one set of rotary files?	3 to 5	41.2
	5 to 10	35.3
	10 to 15	23.5
Can we reuse waste dental burs?	Yes	33.3
	No	43.1
	Don't Know	23.5
How do you sterilize rotary files and burs?	Spirit	35.3
	Autoclave	62.7
	Hot water	2
